# Behavioural abnormalities in a novel mouse model for Silver Russell Syndrome

**DOI:** 10.1093/hmg/ddw357

**Published:** 2016-10-24

**Authors:** Grainne Iseult McNamara, Brittany Ann Davis, Dominic Michael Dwyer, Rosalind M. John, Anthony Roger Isles

**Affiliations:** 1 MRC Centre for Neuropsychiatric Genetics and Genomics, School of Medicine, Cardiff University, Cardiff, UK; 2 School of Psychology, Cardiff University, Cardiff, UK; 3 School of Biosciences, Cardiff University, Cardiff, UK

## Abstract

Silver Russell Syndrome (SRS) syndrome is an imprinting disorder involving low birth weight with complex genetics and diagnostics. Some rare SRS patients carry maternally inherited microduplications spanning the imprinted genes *CDKN1C, PHLDA2, SLC22A18* and *KCNQ1*, suggesting that overexpression of one of more of these genes contributes to the SRS phenotype. While this molecular alteration is very rare, feeding difficulties are a very common feature of this condition. Given that SRS children also have very low body mass index, understanding the underpinning biology of the eating disorder is important, as well as potential co-occurring behavioural alterations. Here, we report that a mouse model of this microduplication exhibits a number of behavioural deficits. The mice had a blunted perception of the palatability of a given foodstuff. This perception may underpin the fussiness with food. We additionally report hypoactivity, unrelated to anxiety or motoric function, and a deficit in the appropriate integration of incoming sensory information. Importantly, using a second genetic model, we were able to attribute all altered behaviours to elevated expression of a single gene, *Cdkn1c*. This is the first report linking elevated *Cdkn1c* to altered behaviour in mice. Importantly, the findings from our study may have relevance for SRS and highlight a potentially underreported aspect of this disorder.

## Introduction

Silver Russell syndrome (SRS) is a very rare genetic disorder with an approximate frequency of 1 in 300,000 (OMIM #180860). The key characteristics that define SRS are foetal growth restriction and failure to thrive postnatally, resulting in a lifelong deficit in height and weight (1–4). While growth restriction is a relatively common generic characteristic that can occur in a number of disorders, SRS patients can be further characterised by some or all of the following: a normal head circumference, which appears large for the body, a large protruding forehead with a triangular-shaped face, clinodactyly, undergrowth of one side of the body (hemihypotrophy), fasting hypoglycaemia, night sweats and excessive thinness which persists into adulthood. A recent clinical scoring system has been proposed for SRS that encompasses a number of these symptoms (4).

The cognitive and behavioural characteristics of SRS are not clear. Some studies indicate normal intelligence and cognitive ability (5–8) while others suggest specific learning difficulties (9–12). There are reports of hyperactivity, emotional problems, conduct problems as well as peer problems with similarities to attention deficit hyperactivity disorder and social communication difficulties with akin to Autism Spectrum Disorders. Although some studies suggest that behavioural problems involving hyperactivity are relatively uncommon (2), one of the first descriptions of SRS reported hyperactivity (13) and one study has reported issues with maintaining attention (11). A more common behavioural observation in SRS centres on feeding issues. Parents of SRS children often express concerns about their child’s eating habits reporting poor appetite, fussy eating and the struggle to get their SRS child to gain weight. Studies reporting poor appetites and/or eating a limited variety of food and/or having issues with food textures reported frequencies of 30% (14), 60% (15), 67% (16) and 82% (17). A recent study using the Netchine-Harbison clinical scoring system reported 100% prevalence of feeding difficulties and/or BMI  < −2SDS in children with a defined SRS (epi)mutation and 92.3% of children meeting the criteria for SRS without a defined (epi)mutations (18) indicating major issues around healthy eating and weight gain.

While some researchers consider SRS a single condition, there are a wide-range of both genetic and epigenetic mutations reported in these patients which may account for differences in their presentation, as suggested in the recent clinical diagnostic scoring system (4). Approximately 70% of patients have alterations affecting human chromosome 7 or 11 while the remaining 30% are of unknown origin (4). Both chromosomes 7 and 11 contain imprinted genes, which are uniquely expressed from either the paternal or maternal chromosome as a consequence of epigenetic marks set up in the male or female germline (19). Some rare SRS patients carry maternal microduplications of 11p15 encompassing *CYCLIN DEPENDENT KINASE INHIBITOR 1C* (*CDKN1C), POTASSIUM CHANNEL, VOLTAGE GATED KQT-LIKE SUBFAMILY Q, MEMBER 1* (*KCNQ1)*, *PLECKSTRIN HOMOLOGY-LIKE DOMAIN, FAMILY A, MEMBER 2* (*PHLDA2)* and *SOLUTE CARRIER FAMILY 22, MEMBER 18* (*SLC22A18)* (20–22). *CDKN1C* is a maternally expressed gene (23,24) and these SRS patients are predicted to have twice the normal level of *CDKN1C* expression (25,26). Mutations in the *CDKN1C* coding region have been observed in some patients with an IMAGe syndrome (MIM 614732), another complex growth restriction disorder whose early clinical features, including IUGR, overlap with that of SRS (27,28). In contrast to the functionally inactivating mutations reported in the congenital overgrowth disorder Beckwith-Wiedemann syndrome (BWS, MIM 130650) (29–33), studies suggest increased protein stability in IMAGe syndrome (28,34) consistent with a role for *CDKN1C* in growth restriction (35).

Loss of *Cdkn1c* has been extensively studied in mouse models, which have highlighted in a role in a number of key features of BWS, including foetal overgrowth and disrupted placental development (36–40). We have recently reported on the phenotype of a mouse, which models the maternal microduplications on 11p15 that are reported in some cases of SRS (41). These mice carry a bacterial artificial chromosome (BAC) transgene providing extra active copies of *Cdkn1c*, *Slc22a18* and *Phlda2* (*Cdkn1c*^BACx1^), are growth restricted *in utero* and remain small throughout their lives (39). In addition, these mice show neonatal hypoglycaemia, head-sparing, a significant loss of white adipose tissue all of which are reported in SRS (41). These mice also possess larger brown adipose tissue depots (41), a phenotype not yet explored in SRS. We were able to attribute all these phenotypes to the just two-fold elevated expression of *Cdkn1c* using a second BAC line of mice that carries exactly the same transgene, but with the transgenic expression of *Cdkn1c* replaced by a *β-galactosidase* reporter gene (*Cdkn1c*^BACLacZ^) (42). Phenotypes present in the *Cdkn1c*^BACx1^ transgenic line and absent in the *Cdkn1c*^BACLacZ^ transgenic line were due to the increased expression of *Cdkn1c*. The presence of some defining SRS features in our unique model supported a role for elevated *CDKN1C* in SRS (41). As we describe here, this model also provides a unique and timely opportunity to assess behaviour in an SRS model, and more specifically in relation to elevated *Cdkn1c*. We found that *Cdkn1c*^BACx1^ animals were hypoactive, had impaired sensorimotor gating and a blunted hedonic response to a palatable solution. Finally, all deficits were rescued by ablation of the additional copy of *Cdkn1c* strengthening a causal relationship between dosage of this gene and potential behavioural abnormalities in SRS.

## Results

### *Cdkn1c*^BACx1^ animals display hypoactivity unrelated to motor dysfunction

**Figure 1.**
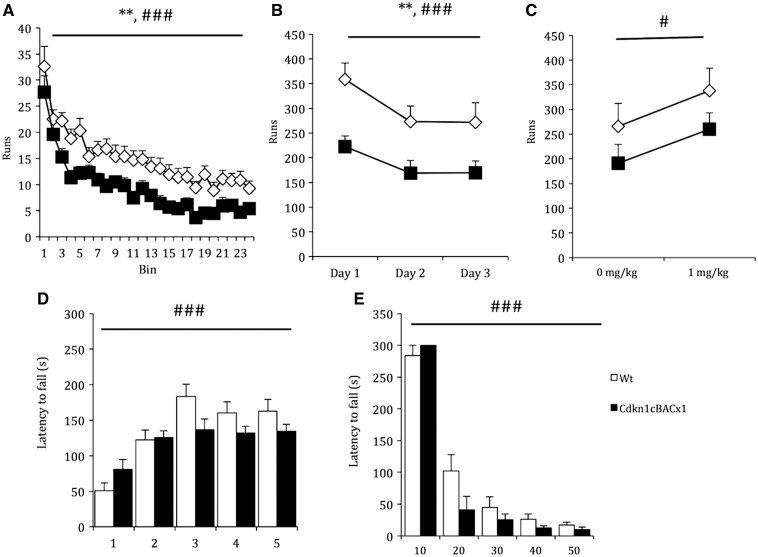
*Cdkn1c*^BACx1^ animals display hypoactivity unrelated to any motoric function deficits. (**A**) Locomotor activity data on the first day of testing broken down into 5-minute bins shows consistent reduced activity of *Cdkn1c*^BACx1^ mice relative to WT littermates throughout the session. (**B**) *Cdkn1c*^BACx1^ also displayed consistent hypoactivity across three consecutive test days. (**C**) All animals showed the expected increase in activity after amphetamine administration. (**D**) Reduced activity in *Cdkn1c*^BACx1^ mice was not related to motoric function deficits as all animals increase time before first fall following successive rotatod training session. (E) Similarly, there was no effect of genotype on latency to fall from an accelerating rotarod across a range of test speeds. Data shown is ± SEM. Main effect of GENOTYPE, *= *P <* 0.05, **= *P <* 0.01; main effect of within subject factors (BIN, SESSION, DOSE, SPEED) #= *P <* 0.05, ###= *P <* 0.001.

In light of reports of hyperactivity in some SRS patients, we first assessed basic motor function in *Cdkn1c*^BACx1^ animals, both in terms of general activity levels and motoric competence. We assayed basal activity for 2-hours on three consecutive days using a standardised general measure where movement is detected by consecutive breaks of two infrared beams (‘run’) (43). *Cdkn1c*^BACx1^ mice were hypoactive relative to their WT littermates, overall making nearly half as many runs in any given session (main effect of GENOTYPE: *F*_1,47 _=_ _8.49, *P* = 0.005) (Fig. 1A and B). This hypoactive phenotype was consistent and not related to a differential reactivity to novelty as, although all animals showed the expected reduction in activity during the first session (Fig. 1A; main effect of BIN, *F*_23,1081 _=_ _38.6, *P <* 0.001) and across the three consecutive days of testing (Fig. 1B; main effect of DAY, *F*_2,94 _=_ _14.6, *P <* 0.001), there was no interaction with GENOTYPE (BIN*GENOTYPE, *F*_23,1081 _=_ _0.61, *P =* 0.92; DAY*GENOTYPE *F*_2,94 _=_ _0.842, *P =* 0.43).

The hypoactivity of *Cdkn1c*^BACx1^ mice was not related to a general impaired motoric function. Firstly, all animals showed the expected increase in number of runs in response to amphetamine (main effect of DRUG: *F*_1,47 _=_ _5.9, *P =* 0.019), and this response was not different by genotype (DRUG*GENOTYPE interaction: *F*_1,47 _=_ _0.01, *P =* 0.94) (Fig. 1C). Secondly, explicit measures of motoric function on the rotarod implied no general motor deficits. Animals were trained to walk on a rotating rod that accelerated across the session duration. All animals learned the task, as indicated by an increase in latency to first fall across five training sessions (main effect of SESSION, *F*_1,27 _=_ _29.1, *P <* 0.001), but there was no main effect of genotype in latency to first fall across the training sessions (main effect of GENOTYPE, *F*_1,27 _=_ _1.92, *P =* 0.18) and no SESSION*GENOTYPE interaction (*F*_4,108 _=_ _1.81, *P =* 0.13). (Fig. 1D). Moreover, during accelerating rotarod probe tests following training, there was no effect of GENOTYPE on latency to fall across five test rotation speeds (main effect of GENOTYPE: *F*_1,27 _=_ _1.47, *P =* 0.24; Fig. 1E).

### Emotional reactivity is not altered in *Cdkn1c*^BACx1^ animals

**Figure 2.**
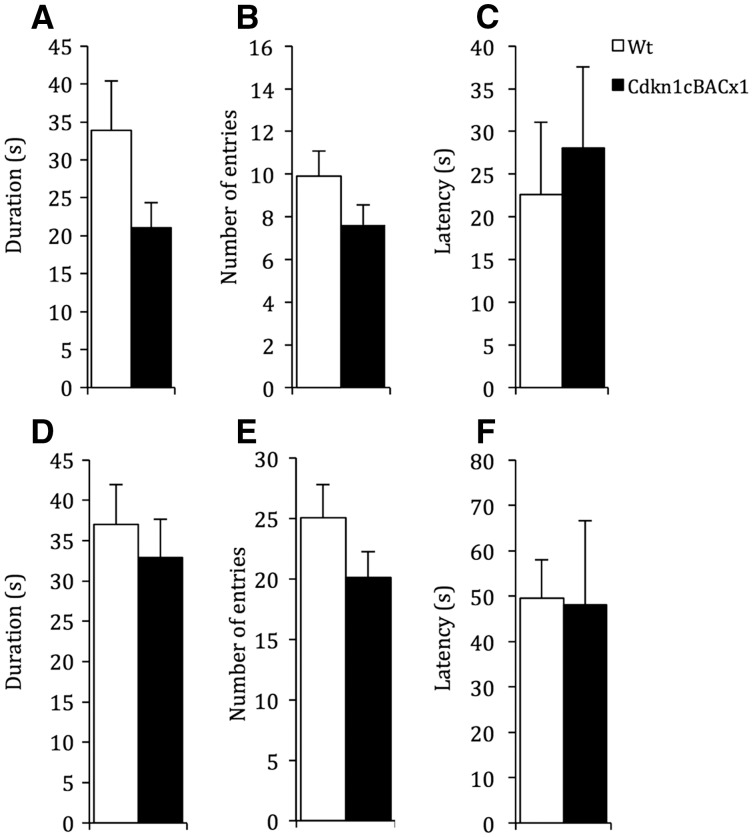
Emotional reactivity was intact in *Cdkn1c*^BACx1^ animals. There were no differences between *Cdkn1c*^BACx1^ animals and their WT littermates in measures on the EPM test, including (**A**) time spent on open arm, (**B**) entries into open arm and (**C**) latency to first enter open arm. Similarly, there were no differences between *Cdkn1c*^BACx1^ animals and their WT littermates in measures on the OF test, including (D) time spent in centre zone, (E) entries into centre zone, and (F) latency to first enter centre zone. Data shown is ± SEM.

Emotional reactivity was assessed using the elevated plus maze test (EPM) and the open field (OF) test. In the EPM, all animals showed the expected pattern of behaviour, spending less time on the anxiogenic open arm than other areas of the maze (middle section, closed arm). Although *Cdkn1c*^BACx1^ animals spent 35% less time on the open arm of the maze on average than their WT littermates (Fig. 2A), this failed to reach significance (t(53)=1.73, *P =* 0.09). A lack of an altered emotional reactivity between *Cdkn1c*^BACx1^ and WT animals on the EPM test was further underlined by no effect of GENOTYPE on number of entries to the open arms (t(53)=1.49, *P =* 0.14) (Fig. 2B) and latency to enter open arms (t(53)=-0.43, *P =* 0.67) (Fig. 2C).

Similarly, the OF test revealed no significant difference between *Cdkn1c*^BACx1^ animals and their WT littermates in the time spent in the anxiogenic inner zone of the arena (t(53)=0.59, *P =* 0.56; Fig. 2D). Again, there was no effect of genotype on number of entries to the inner zone (t(53)=1.41, *P =* 0.16; Fig. 2E) nor in latency to enter open arms (t(53)=0.23, *P =* 0.82; Fig. 2F). These data, from two separate tests, indicate that in emotional reactivity and anxiety behaviours do not differ between *Cdkn1c*^BACx1^ animals and their WT littermates. Consequently, the observed hypoactivity in *Cdkn1c*^BACx1^ was not explained by a difference in the state of anxiety.

### *Cdkn1c*^BACx1^ animals have sensorimotor gating deficits

**Figure 3.**
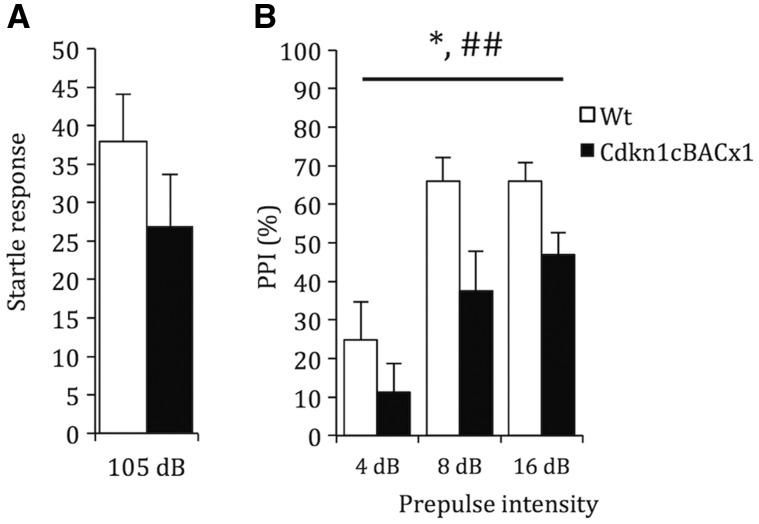
Elevated *Cdkn1c* expression results in sensorimotor gating deficits. (**A**) Average ASR to a 105 dB pulse was not affected by genotype (**B**). However, PPI of the startle response was significantly blunted in *Cdkn1c*^BACx1^ animals. Data shown is ± SEM. Main effect of GENOTYPE, *= *P <* 0.05; main effect of PPI, ##= *P <* 0.01.

Impairments in sensorimotor gating have been described in association with a number of neurodevelopmental disorders including schizophrenia (44) and autism spectrum disorders (45). As like many other studies of mouse models of neuropsychiatric (46) and neurodevelopmental disorders (47), we measured the acoustic startle response (ASR) and the prepulse inhibition (PPI) of the ASR to infer changes in sensorimotor gating here. There was no difference in ASR at 105dB between *Cdkn1c*^BACx1^ and WT littermates (Fig. 3A; t(29)=1.22, *P =* 0.23). However, PPI of the ASR was attenuated by almost half in *Cdkn1c*^BACx1^ relative to WT littermates (Fig. 3B; main effect of GENOTYPE: *F*_1,29 _=_ _6.264, *P =* 0.018). This reduction in PPI in *Cdkn1c*^BACx1^ animals followed the expected pattern of further inhibition with increasing prepulse (main effect of PRE-PULSE: *F*_2,58 _=_ _22.926, *P <* 0.001), and was consistent across the range of pre-pulses used (PREPULSE*GENOTYPE *F*_2,58 _=_ _1.244, *P =* 0.296). This pattern of change with increasing prepulse indicate that *Cdkn1c*^BACx1^ animals are unlikely have a general hearing impairment but that, taken together, these data suggest an impaired sensorimotor gating response relative to WT littermates.

### Impaired perceived palatability in *Cdkn1c*^BACx1^ animals

**Figure 4.**
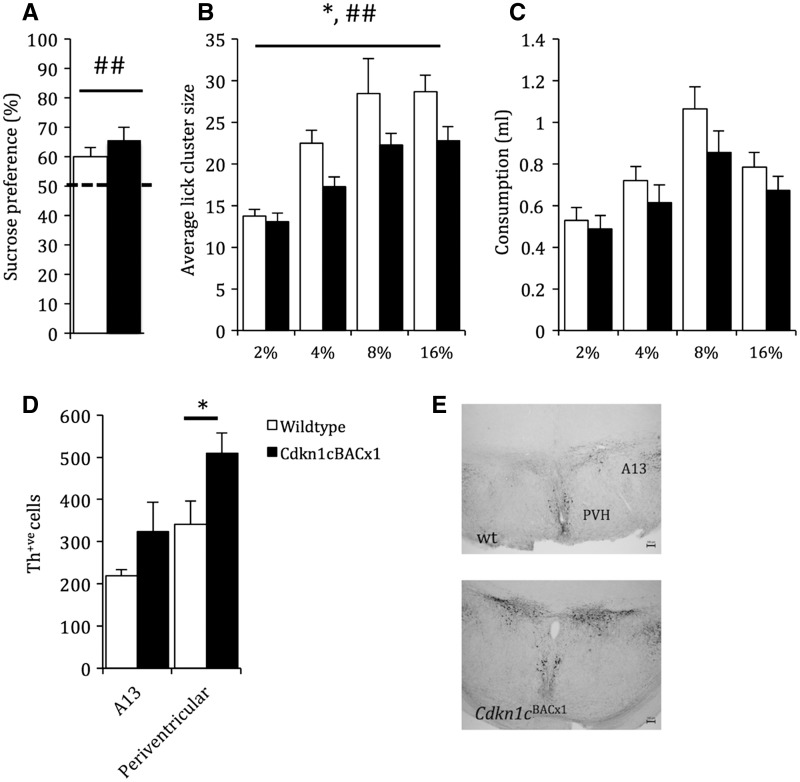
*Cdkn1c*^BACx1^ animals show a reduced perceived palatability of a sucrose solution. (**A**) All animals preferred sucrose to water and this was not affected by genotype. (**B**) *Cdkn1c*^BACx1^ animals had consistently smaller average lick cluster size compared to WT indicating altered hedonic processing. (**C**) Despite potentially reduced perceived palatability, consumption of a palatable solution was not different between *Cdkn1c*^BACx1^ animals and WT. (**D**) *Cdkn1c*^BACx1^ had significantly more Th-positive cells that wt in the periventricular hypothalamus (PVH), but not the A13 dopaminergic cell group. (E) Representative images showing Th staining. Scale bar is 100 μm. Data shown is ± SEM. Main effect of GENOTYPE, *=*P ≤* 0.05; main effect of concentration ##= *P <* 0.01.

Parents of children with SRS often report feeding difficulties with the term "fussy eaters" commonly used to describe this behaviour (15). We previously reported no difference in the consumption of standard chow by *Cdkn1c*^BACx1^ mice (41). In a standard two-bottle preference test, we extend this finding to palatable foods (8% sucrose), showing that both *Cdkn1c*^BACx1^ animals and their WT littermates preferred sucrose over water (Z = 82, *P =* 0.01) (Fig. 4A), but with no effect of genotype (U = 75, *P =* 0.2). However, measures of consumption do not directly provide an indication of the hedonic response to food. To address this, we made use of lick cluster size analysis (LCA). Rodent consumption of a freely available solution is a highly stereotyped behaviour with mice and rats licking in clusters with regular intercluster intervals. LCA can be used as a proxy measure of the perceived palatability of that solution where the average lick cluster size made in response to a palatable solution is related to the hedonic properties of that solution (48). As expected, as the concentration of the sucrose solution increased, there was an overall increase in the average lick cluster size for both groups (Fig. 4B; main effect of CONCENTRATION: *F*_3,162 _=_ _27.1, *P <* 0.001). However, the *Cdkn1c*^BACx1^ mice had a smaller average lick cluster sizes compared to their WT littermates (main effect of GENOTYPE, *F*_1,54 _=_ _5.11, *P =* 0.028), which was steady across all the four sucrose concentrations assessed (CONCENTRATION*GENOTYPE, *F*_3,162 _=_ _1.347, *P =* 0.26). There was no significant difference between *Cdkn1c*^BACx1^ males and their wt littermates in total number licks (main effect of GENOTYPE, *F*_1,54 _=_ _1.281, *P =* 0.26) or in total number of bouts (main effect of GENOTYPE, *F*_1,54 _=_ _0.008, *P =* 0.93). Consistent with other measures of feeding, there was no significant difference overall consumption of the solution (Fig. 4C; main effect of GENOTYPE, *F*_1,54 _=_ _1.515, *P =* 0.224), which excludes an altered satiety threshold underlying the decreased lick cluster size. Critically, there was no effect of genotype on the inter lick interval (ILI; main effect of GENOTYPE: *F*_1,54 _=_ _1.16, *P =* 0.29) (data not shown). ILI is a measure of the mechanical task of the performance. As the ILI was the same in both genotypes, this excludes the difference in licking being as a result of a physical incapacity. Together, these data suggest that the *Cdkn1c*^BACx1^ mice perceived a lower palatability of a sucrose solution compared to WT littermates.

To ask whether the effect of *Cdkn1c* on feeding was associated with alterations in the brain related to feeding and dopamine-signalling, associated with food reward processing in the brain (49), we examined two regions of the hypothalamus involved in the regulation of food intake, the A13 dopaminergic cell group in the zona incerta and periventricular hypothalamus (50–52). Expression of tyrosine hydroxylase (Th), an enzyme involved in the synthesis of dopamine and noradrenaline, was used as a marker of these cell types. There was a significant increase in tyrosine hydroxylase (Th)-positive cells, in the periventricular hypothalamus (t(8)= −2.29, *P =* 0.05) of *Cdkn1c*^BACx1^ with no significant change in the A13 dopaminergic cell group (t(8)=−1.48, *P =* 0.18) (Fig. 4D). This altered neurobiology could contribute to the changes in hedonic behaviour.

### Behavioural deficits in *Cdkn1c*^BACx1^ animals are directly attributable to increased *Cdkn1c* dosage

**Figure 5.**
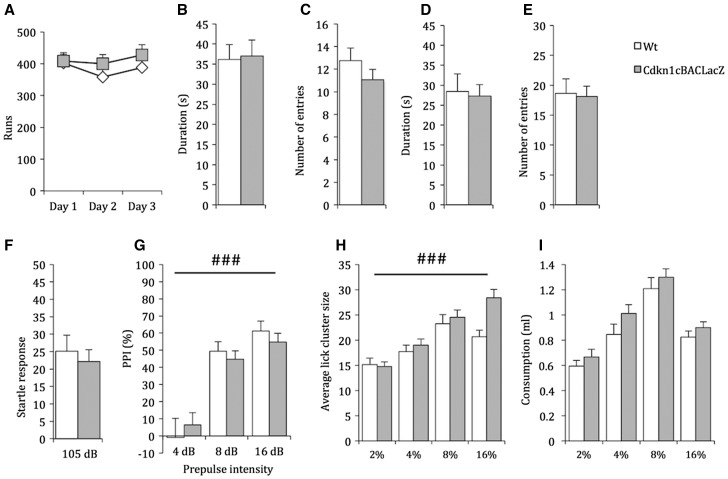
Behavioural alterations in *Cdkn1c*^BACx1^ animals were absent when *Cdkn1c* expression was not elevated. (**A**) There was no difference between *Cdkn1c*^BACLacZ^ animals and their WT littermates in LMA. (**B–E**) Duration of time spent in and number of entries into an anxiogenic zone was not affected by genotype in an (B,C) EPM or (D,E) OF. (**F,G**) ASR and PPI was not different between *Cdkn1c*^BACLacZ^ animals and WT littermates. Average lick cluster size (**H**) was equivalent between groups, as was consumption (**I**) of a palatable sucrose solution. Data shown is ± SEM. Main effects of within subject factors (PPI, Concentration) ###= *P <* 0.001.

The *Cdkn1c*^BACx1^ mouse models the rare 11p15 microduplication seen in a small number of SRS patients (20–22). In this mouse model, as in these rare SRS patients, more than one gene is present within the duplicated region. We wanted to delineate the contribution of these genes, in particular focusing on one, namely *Cdkn1c*, which has a well described role in brain function (53,54). To isolate the contribution of elevated *Cdkn1c* expression to the behavioural phenotypes, we utilized an animal model carrying a single copy of the transgene spanning *Cdkn1c*, but with the transgenic *Cdkn1c* copy ablated. In this model, *Cdkn1c* is expressed at wild type levels (39,41). Critically, none of the behavioural differences found in *Cdkn1c*^BACx1^ were replicated in this model. Specifically, there was no difference in activity between *Cdkn1c*^BACLacZ^ animals and their WT littermates (Fig. 5A; main effect of GENOTYPE: *F*_1,28 _=_ _0.46, *P =* 0.52). There was no difference in time spent in (t(54)=-0.14, *P =* 0.89) or frequency of entries to (t(54)=1.15, *P =* 0.25) the open arms of an EPM (Fig. 5B and C), with convergent findings from the OF test, with no difference in time spent in (t(54)=0.24, *P =* 0.81) or frequency of entries to (t(54)=0.18, *P =* 0.86) the internal zone (Fig. 5D and E). Sensorimotor gating deficits observed in *Cdkn1c*^BACx1^ animals were normal in *Cdkn1c*^BACLacZ^ animals with no difference in ASR at 105 dB (Fig. 5F; t(29)= 0.58, *P =* 0.56) and PPI (Fig. 5G; main effect of GENOTYPE: *F*_1,28 _=_ _2.97, *P =* 0.10). Finally, perceived palatability of a sucrose solution, as measured by LCA, was also equivalent between *Cdkn1c*^BACLacZ^ animals and their WT littermates (Fig. 5H; main effect of GENOTYPE, *F*_1,51 _=_ _2.9, *P =* 0.1) and there was no difference in consumption within the LCA test (Fig. 5I; *F*_1,51 _=_ _2.46, *P =* 0.12).

These data demonstrate that the behavioural abnormalities observed in the *Cdkn1c*^BACx1^ mouse model are attributable specifically to the elevated expression of *Cdkn1c.*

## Discussion

In these experiments we took advantage of a transgenic mouse line that effectively models a rare molecular subtype of patients diagnosed with SRS. These mice carry a single extra copy of a transgene spanning three imprinted genes *Cdkn1c*, *Phlda2* and *Slc22a18* with the minimal duplicated region in SRS spanning one additional gene, *Kcnq1*. We used this model to assess relevant phenotypes concerning activity, emotional reactivity, sensorimotor gating and consumption and response to palatable food. We found selective effects, with the model being generally hypoactive with no obvious deficits in motoric ability, reduced PPI of an ASR and a reduced perceived palatability to sucrose. The extent to which discrete aspects of behaviour could be related to specific gene effects was assessed using a second model in which only *Phlda2* and *Slc22a18* were elevated. The absence of alterations in these specific behaviours in this second model identifies elevated *Cdkn1c* as the causal mutation. In addition to demonstrating the behavioural consequences of increased *Cdkn1c* expression, these data suggest expanding the clinical phenotype assessment of SRS to include more neurological and behavioural measures.

Increased activity has been reported in some patients with SRS, while other studies fail to report an association. Our data on the *Cdkn1c*^BACx1^ model indicated reduced, rather than increased, baseline activity. Although *Cdkn1c*^BACx1^ mice showed the normal reduction in activity over time as they habituated to the apparatus, their general hypoactivity was consistent throughout the first session and across the three consecutive daily sessions. This suggests that this locomotor activity phenotype was not related to any altered reactivity to novelty or increased anxiety. This idea was supported by data from explicit tests of emotional reactivity, the elevated plus maze and open field, where there were no differences in behaviour between *Cdkn1c*^BACx1^ mice and their WT counterparts. Furthermore, the reduced activity was not due to a physical impairment, as *Cdkn1c*^BACx1^ mice increased their locomotor activity in a similar pattern to WT after acute amphetamine administration and also showed no difference in motor learning and skill on the rotarod test. Reports of hyperactivity in individuals with SRS are infrequent, however, why there should be a discrepancy between reports of hyperactivity in some SRS patients and robust evidence of hypoactivity in the *Cdkn1c*^BACx1^ SRS mouse model is not clear. This may relate to reporting (as opposed to explicit measures of activity) and/or whether the genetic sub-types of SRS have been taken into account.

Sensorimotor gating, as measured here by PPI of the ASR, provides a measure of appropriate integration of sensory cues with a motor output. Although we found no difference in the response to an acoustic startle, *Cdkn1c*^BACx1^ mice did show a reduced PPI. *Cdkn1c*^BACx1^ mice did show the expected increase in PPI of startle in response to an increasingly loud pre-pulse tone, but there was a general reduction in the degree of inhibition. This suggests the reduced PPI is not due to hearing problems in the *Cdkn1c*^BACx1^ mice, but is more likely to be related to specific deficits in sensorimotor gating. This pattern of abnormalities is seen in a number of psychiatric disorders (44) and also autism spectrum disorders (45). Although the incidence of psychiatric illness in SRS is very rare (55), there are a growing number of studies beginning to link SRS with neurodevelopmental disorders, such as autism spectrum disorders (56).

One of the more widely reported behaviours in SRS relates to difficulties with food; fussiness with regards to food, and children who are simply not interested in food. Since a key complication in SRS is the failure to gain weight and extreme skinniness, it is important to understand the biology underpinning this behaviour. We previously reported that *Cdkn1c*^BACx1^ mice possess more energy consuming brown adipose tissue but consumed the same amount of standard chow as their littermates over a 7-day measurement (41). This could account for the low BMI reported in SRS but would not explain fussy eating. Here, we show that consumption of a palatable sucrose solution was equivalent between *Cdkn1c*^BACx1^ and WT littermates. However, consumption does not provide a measurement of the hedonic response to food. In order to test this, we made use of LCA, a behavioural measure of perceived palatability (48) used previously to examine rodent models of psychiatric disorders (57) and the imprinting disorder, Prader Willi Syndrome (58)*.* Rodents rarely show continuous consumption of a liquid. Instead they perform repeated clusters of licks separated by pauses. The number of licks in each cluster (cluster size) has a positive, monotonic relationship with the concentration of a palatable solution such as sucrose (59). Importantly, cluster size is not simply a reflection of the amount consumed. For example, although the cluster size increases with increased sucrose concentration, the amount consumed varies in an inverted U-shaped function. In addition, studies of conditioning have also shown that cluster size measures of hedonic reactions and consumption measures can dissociate (60). Therefore, the cluster size is a sensitive measure of the palatability of a solution. Here we show that, although *Cdkn1c*^BACx1^ mice consumed the same volume of a sucrose solution, they did so in smaller lick clusters. This indicates that perceived palatability in *Cdkn1c*^BACx1^ mice is reduced relative to WT littermates; for instance *Cdkn1c*^BACx1^ mice have a similar lick cluster size at 8% sucrose as WT mice at 4% sucrose (Fig. 4B). This would suggest perturbed food reward processing in the *Cdkn1c*^BACx1^ mice, with effects on the perceived hedonic value of food. In human studies, it has been demonstrated that there is a positive relationship between food palatability and amount consumed (61). In human SRS patients, it is easy to imagine how a lowered hedonic response may translate into fussiness or a disinterest in food and, consequently, reduced food intake.

One of the complexities of studying any phenotype associated with an imprinting disorder, such as SRS, is the potential involvement of multiple genes. As previously (41), we were able to address this issue using a separate control line of transgenic mice carrying the same BAC transgene, but without elevated expression of *Cdkn1c* (*Cdkn1c*^BACLacZ^). The behaviour of the *Cdkn1c*^BACLacZ^ mice was equivalent to WT in all of the behavioural tests described here. These data implicate over-expression of *Cdkn1c* as the critical molecular factor underlying the hypoactivity, reduced PPI and lowered hedonic response to sucrose seen in the SRS model, the *Cdkn1c*^BACx1^ mice. This is perhaps unsurprising, given the minimal brain expression of the other BAC genes, *Phlda2* and *Slc22a18*. In contrast, *Cdkn1c* has a key role in the developing nervous system through the regulation of cell cycle exit, differentiation and migration of embryonic cerebral cortical precursors (54), with loss-of-function resulting in an increased thickness of layer six of the cortex during development (62). In addition to early development, *Cdkn1c* has also been shown to be key to the regeneration of the adult brain through regulating adult neural stem cell quiescence (63). The functional relevance of this role in adult neural stem cells has not been explored in relation to behaviour but there may be further influences on behaviour as the mice age or under specific conditions where appropriate neurogenesis is required e.g. learning and memory (64). However, possibly most pertinent to the behaviours outlined here is the role of *Cdkn1c* in the developing dopaminergic system. For instance, *Cdkn1c* co-expression with *Nurr1* promotes maturation of a midbrain dopaminergic neuronal cell line while loss-of-function *in vivo* results in the failure of this cell types to differentiate in a timely manner (53). Perturbation of the dopamine systems has long been linked to changes in locomotor activity (65,66) and sensorimotor gating (67,68). Dopamine signalling is also associated with food reward processing in the brain (49). We show an increase in the number of Th-positive cells in the A13 dopaminergic cell group and periventricular hypothalamus. As Th is marker for both dopaminergic and noradrenergic neurons, and the observation that *Cdkn1c* is involved in embryonic dopaminergic cell proliferation, this indicates that the development of the dopaminergic system may be compromised in mice over-expressing *Cdkn1c*. Moreover, given the role of the hypothalamus in **the** regulation of food intake (50–52), the increased number of Th-positive cells in these regions may contribute to this phenotype.

Patients with microduplications spanning *CDKN1C* are very rare (20–22). The majority of SRS patients do not carry genetic mutations affecting *CDKN1C* or epigenetic alterations at the imprinting centre for *CDKN1C* (*KCNQ1OT1:TSS-DMR)*, which lies over 200 kb away from the gene (69,70). A key question that this study raises, therefore, is the extent to which elevated *CDKN1C* contributes more widely to SRS. Loss of paternal silencing in mice can occur in circumstances where DNA methylation at the imprinting centre is intact. For example, when the non-coding RNA *Kcnq1ot1/Lit1* is prematurely terminated (71,72), when DNA methylation is not maintained in the somatic DMR in the promotor of *Cdkn1c* (73–75) and when repressive histone marks are not propagated (76). Therefore biallelic expression of *CDKN1C* in humans could occur independent to changes in *KCNQ1OT1:TSS-DMR* methylation. *CDKN1C* levels have not been extensively examined in patients with (epi)mutations at the locus 11p15 locus or in those 40% of patients without an identified genetic or epigenetic defect. Further work is required to establish the prevalence of elevated *CDKN1C* in SRS and the extent to which our behavioural observations may inform the assessment of these patients. Nonetheless, there is now a considerable body of evidence from animal models and human studies to indicate a role for the imprinted *CDKN1C* gene in at least three very rare disorders affecting foetal growth (SRS, BWS and IMAGe) syndrome. Importantly, the new knowledge presented in this study will contribute to an improved understanding of the long-term behavioural implications for all these disorders.

In summary, we present the first characterisation of rodent behaviour in response to elevated expression of the imprinted *Cdkn1c* gene, which identified alteration in in locomotor activity, sensori-motor gating and the hedonic response to food. Although we do not characterise the specific neural perturbation in this model in detail, we observed alterations in a number of Th-positive neurons in the hypothalamus suggesting a direct effect on the developing central nervous system. Further work is required to clarify the relationship between elevated *CDKN1C* and SRS with a specific reference to neurodevelopmental problems. Indeed, different genetic sub-types of SRS may present with differing ranges of additional problems, as has been suggested previously with respect to SRS scoring system (4). Our data strongly suggest that this would be an avenue of research worth exploring. A better understanding of the pathobiology underpinning any behavioural and cognitive abnormalities, and food disorders in particular, in SRS patients will be helpful in the design of therapeutic approaches in the longer term.

## Materials and Methods

### Animals

The experimental line *Cdkn1c*^BACx1^ possesses one copy of a bacterial artificial chromosome that spans the *Cdkn1c* gene and two other genes, *Phlda2* and *Slc22a18*. The reporter line *Cdkn1c*^BACLacZ^ possesses a modified version of this BAC with a *β-galactosidase* reporter construct inserted into the *Cdkn1c* locus, disrupting *Cdkn1c* expression (27). *Cdkn1c*^BACLacZ^ functions as a control for the experimental line to attribute any phenotypes observed specifically to the over expression of *Cdkn1c* (i.e. present in line *Cdkn1c*^BACx1^ and absent in line *Cdkn1c*^BACLacZ^). Both lines were maintained on a C57BL/6 background having been bred onto this background for >12 generations. One hundred and twenty-nine male mice were used for behavioural assessment: *Cdkn1c*^BACx1^ (*n =* 40), WT littermates (*n =* 36), *Cdkn1c*^BACLacZ^ (*n =* 31) WT littermates (*n =* 22). For all testing minimum group size per genotype was 13. Degrees of freedom is stated in all statistical analysis reflecting the total group size.

### LMA and motoric function

LMA behaviour was tested using as described (43) with two infra-red beams crossing each cage 30mm from each end and 10mm from the floor of the chamber. Beam breaks were recorded as an indication of activity, using a computer running custom written BBC Basic V6 programmes with additional interfacing by ARACHNID (Cambridge Cognition Ltd, Cambridge, U.K.). Data stored were the total number of beam-breaks in a 2 hour session. One session was carried out per day, for three consecutive days as a measure of habituation to a novel environment. Habituation to a novel arena was assayed by the decrease in beam breaks across sessions.

Following the three days of basal locomotor activity testing, animals were injected intra-peritoneally (i.p.) with saline or 1 mg/kg of D-amphetmine sulphate (Tocris Bioscience, Bristol, UK). All animals received each solution in a pseudo-randomised order, with 72 hours between each injection to allow for solution wash out. Session total consecutive beam breaks (runs) were used as an indication of amphetamine induced LMA. A rotarod task (Ugo Basile, Italy) was used to assess motoric function. Animals received five training sessions. In the test session latency to fall at a set rotating speed was recorded.

### Emotional reactivity

#### EPM

Anxiety was measured using an EPM. Briefly, apparatus, consisting of four Perspex arms two open (175 × 78 mm) and two enclosed (190 × 80 × 150 mm) with an open roof, was used. Animals were placed centrally at the beginning of a trial and allowed to explore freely for 300 s. Activity was tracked using ETHOVISION software (Noldus, Nottingham, UK). Increased time spent in the open arm was considered less anxious behaviour.

#### OF test

Locomotor activity and anxiety were measured using distance moved in, and time spent in the centre of, respectively, an OF. Briefly, an OF apparatus (750 × 750 mm) was used, divided into 2 virtual zones, an inner (central 450 × 450 mm) and an outer (150 mm periphery). Animals were allowed to explore the arena freely for 600 s and activity was tracked using ETHOVISION software (Noldus, Nottingham, UK). Greater time spent in the inner zone was considered less anxious behaviour.

### ASR and PPI

ASR and PPI were monitored using a SR-Lab apparatus (San Diego Instruments, U.S.A) modified for use in mice. Animals were placed in a Perspex tube (internal diameter 35mm) and white noise stimuli were presented via a speaker. Pulse-alone trials consisted of a 40ms startle stimulus and a prepulse trial consisted of a 20ms prepulse at 4, 8, or 16db above background and a 40ms 105db startle stimulus, 70ms after the prepulse. The stimuli were presented in a pseudorandom manner every 15s. Whole body startle responses were recorded as the average startle during a 65ms window timed from the onset of the startle pulse. PPI was calculated as the percentage reduction in startle between prepulse trials and pulse alone trials.

### Preference testing

Mice had restricted food access for the duration of testing, having no access to food for 16 hours prior to testing. All testing took place in opaque Perspex boxes with access to the solution provided for thirty minutes, once per day. Preference testing was carried out on seven consecutive days. For three days mice were presented with two bottles of water to ensure there was no side bias. For the following four consecutive days one bottle contained an 8% (w/v) sucrose solution, which altered left-right presentation by day. Bottles were weighed before and after testing and sucrose preference was calculated as (volume sucrose consumed/total volume consumed)*100.

### Lick cluster analysis

LCA testing was carried out as described (58) with food restriction as in preference testing. A contact sensitive lickometer registered each lick to the nearest 0.01 s, recorded using MED-PC software (Med Associates Inc., St. Albans, VT, USA). Animals were trained to drink 8% (w/w) sucrose in water prior to testing, until consumption had stabilised (requiring 4–14 sessions). Animals were presented with a solution of a given sucrose concentration for a minimum of four days; the order of presentation was counterbalanced for genotypes. Sessions in which data records indicated errors in lick recording or spout blockage were removed from the analysis; specifically recording fewer than 10 bouts, greater than 40 isolated single licks or average interlick intervals of <90 or >250 ms. This equated to the exclusion of 48/721 data points for *Cdkn1c*^BACx1^ animals and WT littermates and 117/752 data points for *Cdkn1c*^BACLacZ^ animals. Inclusion of these data points does not alter the result. The data from the first day at each new concentration was excluded. Data were averaged across sessions at each concentration for comparison. A pause between licks of greater than 0.5 s was defined as a new cluster.

### Immunohistochemistry

Animals were transcardially perfused with 10% formalin fixative (Sigma-Aldrich, UK) and brains removed. Following post-fixing overnight, brains were removed to a solution of PBS containing 30% sucrose. 40 μm sections were obtained from Bregma +2 to −2.5 on a freezing microtome. Immunostaining for Th was carried out on 1 in 5 sections (every 120 μm). Free-floating sections were incubated overnight in 1:1000 rabbit-α-Th (Abcam, UK) and section was performed using standard DAB staining (Vector labs). Number of positive cells in A13 dopamnergic cell group of the zona incerta and periventricular hypothalamus were counted on all sections.

### Statistics

All statistical analyses were carried out using SPSS 20.0 (SPSS, USA). Simple effects were analysed by Students t-test. For repeated behavioural analysis a series of ANOVAs were carried out on the data with a between subject factor of GENOTYPE and within subjects factor of CONCENTRATION/DAY/BIN/PRE-PULSE INTENSITY, where appropriate. Sucrose preference data and effect of genotype on preference were analysed using Wilcoxon signed rank and Mann-Whitney U-tests, respectively. Differences in Th immunoreactivity was analysed by Students t-test.
